# Dynamic Characteristics of Vertically Coupled Structures and the Design of a Decoupled Micro Gyroscope

**DOI:** 10.3390/s8063706

**Published:** 2008-06-03

**Authors:** Bumkyoo Choi, Seung-Yop Lee, Taekhyun Kim, Seog Soon Baek

**Affiliations:** Department of Mechanical Engineering Sogang University / #1 Shinsu-dong, Mapo-gu, Seoul 121-742, Korea

**Keywords:** vibratory gyroscope, coupled motion, vibration analysis

## Abstract

In a vertical type, vibratory gyroscope, the coupled motion between reference (driving) and sensing vibrations causes the zero-point output, which is the unwanted sensing vibration without angular velocity. This structural coupling leads to an inherent discrepancy between the natural frequencies of the reference and the sensing oscillations, causing curve veering in frequency loci. The coupled motion deteriorates sensing performance and dynamic stability. In this paper, the dynamic characteristics associated with the coupling phenomenon are theoretically analyzed. The effects of reference frequency and coupling factor on the rotational direction and amplitude of elliptic oscillation are determined. Based on the analytical studies on the coupling effects, we propose and fabricate a vertically decoupled vibratory gyroscope with the frequency matching.

## 1. Introduction

Recently, there is increasing needs of the micro gyroscope in the commercial application filed, such as the automobile, the camcorder, the VR HMD set (Virtual reality head mount display), and so on. Though the many researchers have tried to develop the reliable and low-cost gyroscope, it is not commercially available yet because there are some problems, such as the exact frequency tuning, the wafer level vacuum packaging, and the temperature dependency on the structure material [1-3].

The vertical-type gyroscopes, in which the reference and the sensing directions are perpendicular, become main streams in micro-scale sensors. In the resonant vibratory gyroscope, the sensitivity is maximized when the driving and the sensing frequencies are exactly tuned. However, the driving and the sensing frequencies of the fabricated gyroscope are barely same due to the mechanical tolerance and fabrication error, even though the gyroscope has the exactly same frequencies in design stage. On the other hand, the coupled gyroscope that uses the same spring in the driving and the sensing modes shows mode coupling effect when the difference between the driving and the sensing mode frequencies is smaller than 100 Hz[4]. The coupled motion between the reference and the sensing vibrations causes the zero-point output, which means non-zero sensing vibration without angular velocity. Then, the gyroscope makes the vibration motion of the elliptic trajectory[5]. The understanding of the coupled motion in terms of the dynamic characteristics would be essential and the design of decoupled structure should be required based on it. To decrease the mechanical coupling of vibrating gyroscopes, various structures have been proposed where the drive and sense motions are independent [5-7].

In this paper, we examine the dynamic characteristics of zero-point output induced by the structural coupling in the vibratory gyroscopes, and propose a new vertical decoupled gyroscope with the exact frequency matching based on the theoretical analysis on the coupling phenomenon.

## 2. Vibration of a vertically coupled structure

**Figure 1.**
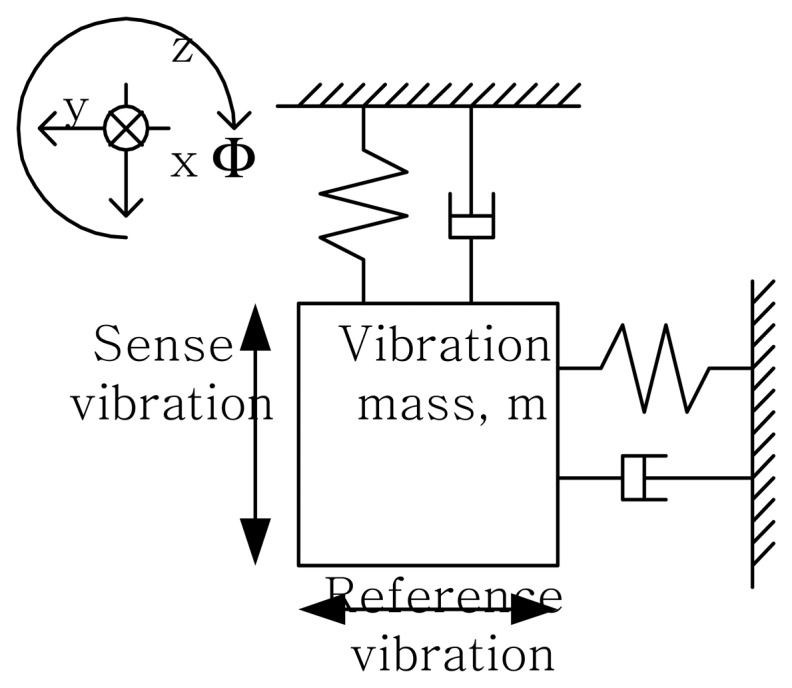
Modeling of Microgyroscope.

The vertical gyroscope can be modeled as a 2-degree of freedom vibration system shown in Fig. 1. The mass of the gyroscope is harmonically driven along the y-axis. This oscillation is referred as the reference (driving) vibration. When the angular velocity, Φ, in the *x* direction about *z* axis is applied to the system, the oscillation in the *x* direction, *x*(*t*), should be generated by a Coriolis acceleration. The amplitude of this oscillation (sensing vibration), which is proportional to the angular velocity, is measured by evaluating the capacitance changes between the mass and the bottom electrode.

Unwanted vibration occurs through the structural coupling between the reference and the sensing motions of a vibratory gyroscope. The structural coupling comes mainly due to inherent fabrication errors, residual stresses and material properties, and it is the main source to deteriorate the sensing performance of the gyroscope. In particular, since the reference frequency is set to resonant frequency in order to increase the sensitivity of the sensor, the mechanical coupling intensifies the zero-point output.

### 2.1. Modeling and vibration analysis of the coupled structure

**Figure 2.**
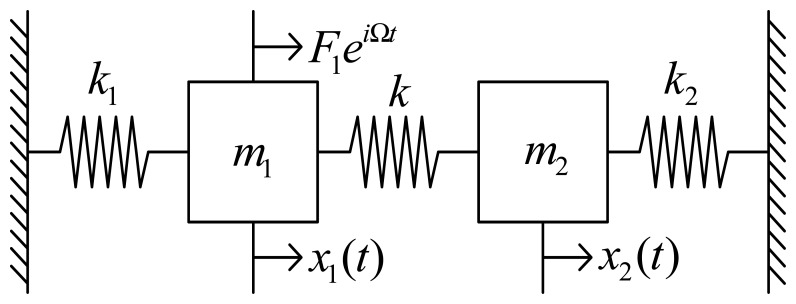
Modeling of the coupled structure.

As shown in Fig. 2, we introduce a simplified model to analyze dynamic characteristics of the vertically coupled gyroscope. Here *x*_1_(*t*) and *x*_2_ (*t*) are the reference and the sensing motions. *k* is the stiffness coefficient to quantify the degree of the structural coupling. *F*_1_ and Ω are the amplitude and the exciting frequency of the external force applied to the reference motion. The equation of motion of the coupled system is

(1) $$\left[\begin{array}{cc} m & 0\\ 0 & m \end{array}\right]\;\left[\begin{array}{c} {\ddot{x}}_{1}\\ {\ddot{x}}_{2} \end{array}\right]+\left[\begin{array}{cc} k_{1}+k & -k\\ -k & k_{2}+k \end{array}\right]\;\left[\begin{array}{c} x_{1}\\ x_{2} \end{array}\right]=\left[\begin{array}{c} F_{1}\\ 0 \end{array}\right]\;e^{i\Omega t}$$

To analyze free vibration of the system, we use **x** (*t*) = [*x*_1_ *x*_2_]*^T^* = **u** *e^iω_n_t^* and introduce the following parameters

$$\begin{array}{ccc} r^{2}=\frac{k}{k_{1}}, & k_{12}=\frac{k_{2}}{k_{1}}, & \lambda =\frac{\omega_{n}^{2}m}{k_{1}}\;. \end{array}$$

Finally, the normalized eigenvalue problem is given by

(2) R u=λu

where $\text{R}=\left[\begin{array}{cc} 1+r^{2} & -r^{2}\\ -r^{2} & k_{12}+r^{2} \end{array}\right]=\left[\begin{array}{cc} R_{11} & R_{12}\\ R_{21} & R_{22} \end{array}\right]$ and *r*^2^ is the coupling strength and *λ* is the normalized natural frequency.

Eigenvalues for the decoupled case (*r*^2^ = 0) are *λ*_1_ = 1 and *λ*_2_ = *k*_12_. With the coupling effect, the eigenvalues, *λ*_1_ = 1 and *λ*_2_ = 1 + 2*r*^2^, are obtained from Eq. (2) by assuming *k*_12_ = 1.

### 2.2. Curve crossing and veering in frequency loci

**Figure 3.**
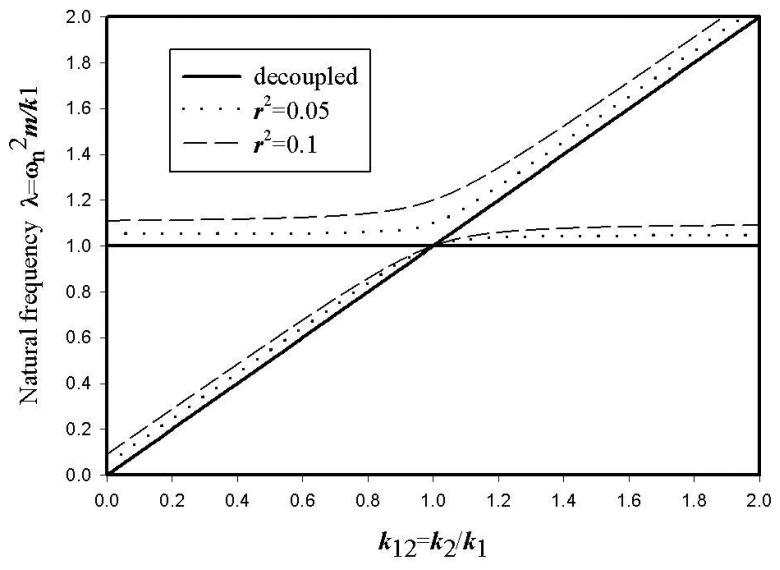
Curve crossing and veering in frequency trajectories.

When the natural frequencies (or eigenvalues) of a coupled system are frequently plotted as a function of a coupling parameter, two frequency loci approach each other and then they often cross or abruptly diverge. The former case is called “curve crossing” and the latter one “curve veering”[9]. Whether the two converging loci intersect or not, strongly depends on dynamic characteristics of the coupled system. Figure 3 shows the normalized natural frequencies of the reference and the sensing motions of the coupled system given by Eq. (2) versus the stiffness ratio *k*_2_/*k*_1_. For the decoupled case (*r* = 0), two frequency curves cross each other. However, the curve veering around the frequency*λ* = 1 if the structural coupling (*r* ≠ 0) occurs. The strength of the curve veering depends on the degree of the structural coupling. In a vibratory gyroscope, the curve crossing between the reference and the sensing frequencies should be achieved in order to increase the sensing performance. However, it is noted that it is impossible to achieve the exact frequency matching for the structurally coupled gyroscope.

### 2.3. Forced vibration analysis

To analyze dynamic characteristics of zero-point out, which is generated by the structural coupling between the reference and the sensing vibrations, we solve the forced response to harmonic excitation. The substitution of **x** (*t*) = ***X*** *e^i^*^Ω^*^t^* into Eq. (1) gives the amplitudes ***X*** = [*X*_1_ *X*_2_]*^T^* of the reference and the sensing vibration

(3) $$\frac{X_{1}}{F_{1}}=\frac{k_{2}+k-\Omega^{2}m}{(k_{1}+k-\Omega^{2}m)(k_{2}+k-\Omega^{2}m)-k^{2}}$$

(4) $$\frac{X_{2}}{F_{1}}=\frac{k}{(k_{1}+k-\Omega^{2}m)(k_{2}+k-\Omega^{2}m)-k^{2}}$$

The following parameters are introduced to normalize the forced response.

$$\begin{array}{ccc} r^{2}=\frac{k}{k_{1}}, & \omega^{2}=\frac{\Omega^{2}m}{k_{1}}, & k_{12}=\frac{k_{2}}{k_{1}} \end{array}$$

Here *r^2^* is the degree of the coupling, *k*_12_ the ratio of *k*_1_ to *k*_2_, and *ω*^2^ the reference frequency. Since the reference motion *x*_1_(*t*) is vertical to the sensing motion *x*_2_(*t*), the actual motion of the gyroscope is represented by the following complex form *x*_1_ + *ix*_2_. Then the coupled motion becomes

(5) $$\frac{x_{1}^{2}}{{\left|X_{1}\right|}^{2}}+\frac{x_{2}^{2}}{{\left|X_{2}\right|}^{2}}=F_{1}^{2}$$

Thus, the zero-point output of the vertically coupled vibratory gyroscope has the elliptic motion.

### 2.4. Shape of the zero-point output corresponding to the reference frequency

Consider a gyroscope with *k*_12_ = 1 and *F*_1_ = 1 for the simplicity of calculation. The amplitudes of *x*_1_(*t*) and *x*_2_(*t*) are from (3) and (4)

(6) $$X_{1}=\frac{1+r^{2}-\omega^{2}}{\left(1+2r^{2}-\omega^{2})(1-\omega^{2}\right)}$$

(7) $$X_{2}=\frac{r^{2}}{\left(1+2r^{2}-\omega^{2})(1-\omega^{2}\right)}$$

**Figure 4.**
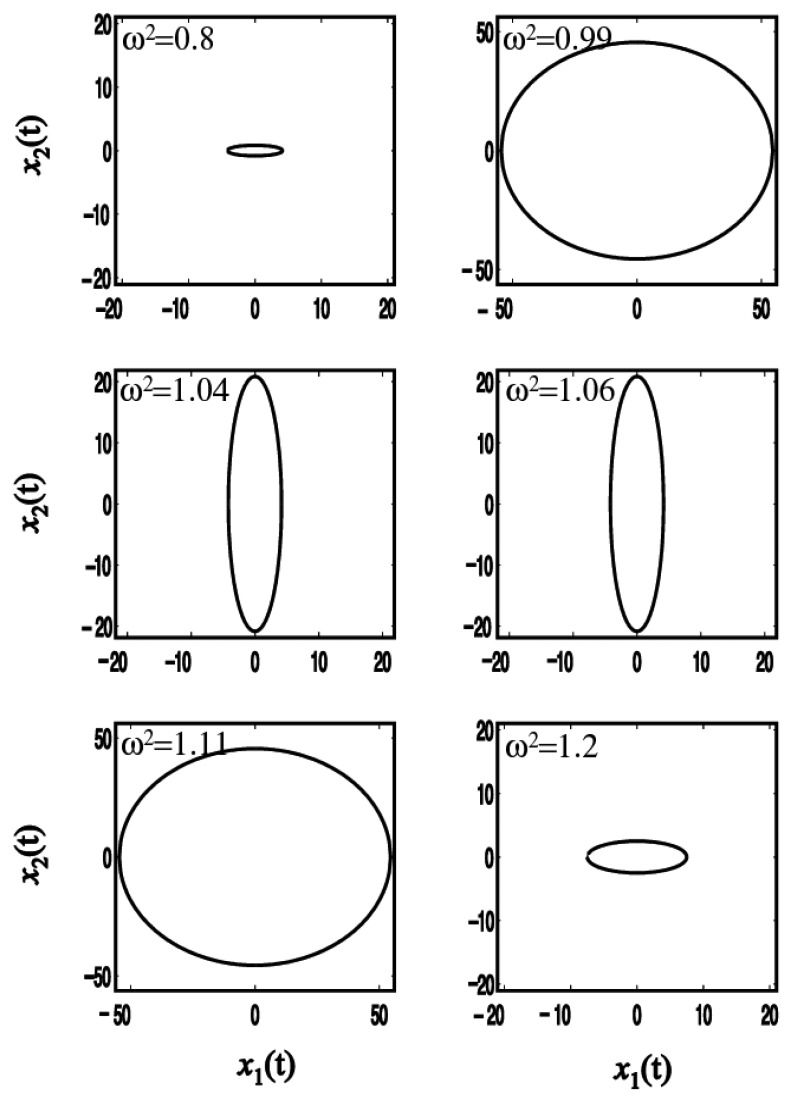
Simulation of zero-point output depending on reference frequency.

The zero-point output is plotted as a function of the reference frequency *ω* when *r*^2^ = 0.05 in Fig. 4. When the system is excited at the frequencies below the first natural frequency, 1, or above the second one, $\sqrt{1+2r^{2}}$, the reference vibration is larger than the sensing one resulting in an ellipse with the major axis *x*_1_. As the excitation frequency is close to the resonance one, the elliptic motion is changed to a circle. The radius of the circle becomes infinite at resonance. When the forced frequency is between the first and second frequencies, the forced response is shown to have an ellipse with a large sensing vibration. It is noted that the reference vibration *x*_1_ would be zero and only the sensing motion occurs at *ω*^2^ = 1 + *r*^2^.

**Figure 5.**
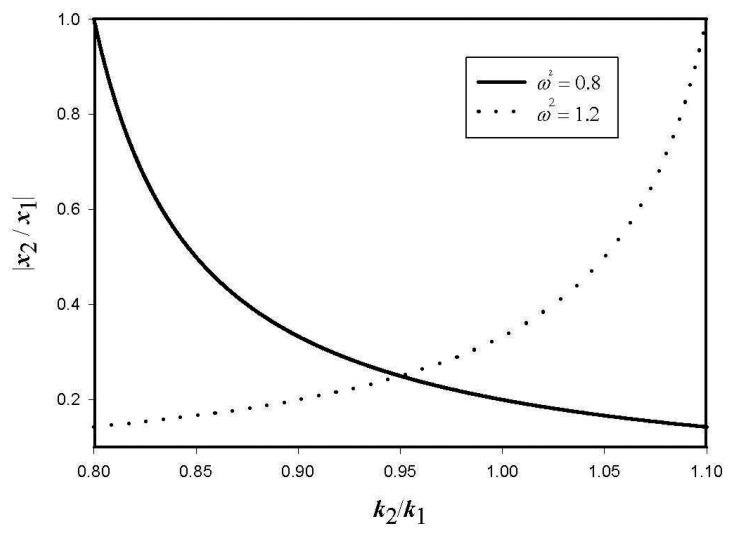
Amplitude ratio vs. stiffness ratio.

Figure 5 shows the amplitude ratio of the reference and the sensing vibrations as a function of *k*_12_ = *k*_1_/*k*_2_ for two cases. When the excitation frequency is less than the first natural frequency (*ω*^2^ = 0.8), the sensing vibration by the coupling effect becomes smaller for larger *k*_12_. However, as the system is excited above the second natural frequency (*ω*^2^ = 1.2>1 + 2*r*^2^), the coupling effect increases for increasing *k*_12_. Note that the degree of the coupling effect can be adjusted by changing the ratio of *k*_1_ and *k*_2_but the sensitivity of the system is reduced at *k_1_* ≠ *k*_2_. Therefore, both effects of the structural coupling and sensitivity should be considered simultaneously in designing the gyroscope.

### 2.5. Rotational direction of the coupled motion

From Eq. (5), the zero-point output induced by structural coupling is shown to have an elliptic motion. The rotational direction of the elliptic motion as a function of the driving frequency can be determined as follows: Firstly, introducing the complex displacement

(8) $$z(t)=x_{1}(t)+ix_{2}(t),$$

we denote Eq. (3) as

(9) $$m\ddot{z}+k^{*}z+\Delta k^{*}\bar{z}-ki\bar{z}=\frac{F_{1}}{2}(e^{i\Omega t}+e^{-i\Omega t})$$

where $k^{*}=\frac{k_{1}+k_{2}}{2}+k$ and $\Delta k^{*}=\frac{k_{1}-k_{2}}{2}-ki$. Here *z̅*(*t*) is the complex conjugate of *z*(*t*). Since for most gyroscopes *k*_1_ = *k*_2_ the parameters become k* = *k*_1_ + *k* and Δk* = −*ki*. Then, the solution of Eq. (9) can be expressed by the forward and backward directions.

(10) $$z(t)=z_{f}\;e^{i\Omega t}+z_{b}e^{-i\Omega t}$$

The substitution of (10) into (9) gives the amplitudes of the forward and backward whirls, respectively.

(11) $$z_{f}=\frac{1}{2}\frac{\left(r^{2}+1-\omega^{2}+2r^{2}i\right)}{\left(r^{2}+1-\omega^{2}+r^{2}i)(-m\Omega^{2}+k_{1}+k\right)}$$

(12) $${\bar{z}}_{b}=-\frac{1}{2}\frac{1}{\Delta k^{*}-m\Omega^{2}+k^{*}}=\frac{1}{2k_{1}(-r^{2}-1+\omega^{2}-r^{2}i)}$$

We can determine the direction of the elliptic motion by checking the amplitude ratio of the forward and backward components

(13) $$\left|\frac{z_{f}}{{\bar{z}}_{b}}\right|=\frac{\sqrt{{\left(r^{2}+1-\omega^{2}\right)}^{2}+4r^{2}}}{\left|\omega^{2}-1+r^{2}\right|}.$$

When this ratio is greater than unity, the system rotates in the same direction (counter clockwise) of the reference vibration. If it is below 1, the system rotates in the backward direction (clockwise). The reference frequency that makes the value of Eq. (13) unity should be *ω*^2^ =1 + *r*^2^. Therefore, the rotational direction turns from counter clockwise to clockwise for the frequency of 1+ *r*^2^ which is the midpoint between the two natural frequencies of 1 and 1+ 2*r*^2^. Table 1 shows the shapes and the rotating directions corresponding to the reference frequency.

### 2.6. Quantification of the coupling degree

The degree of coupling *r*^2^ can be derived from the eq. (7) as following.

(14) $$r^{2}=\frac{-X_{2}(1-\omega^{2}+\omega^{4})}{2X_{2}(1-\omega^{2})-1}$$

From the above expression, the degree of structural coupling *r*^2^ would be quantified by measuring the amplitude of the sensing motion *x*_2_. This relationship could be utilized in the optimization of the various designs for a decoupled structure and it is useful to predict the zero-point output for the reference frequency without experiments of trial and error. In order to make the structure decoupled, we should reduce the degree of coupling, *r*, to zero ideally. Also, we know that the structure should have the unconstrained spring so as to make the degree, *r*, zero.

## 3. Design of a new decoupled gyroscope

A vibratory gyroscope is based on a pair of the driving and the sensing resonators. In order to increase the sensitivity of the vibratory gyroscope, the driving and the sensing frequencies should be exactly tuned. However, a gyroscope having the inherent coupled structure shows unwanted sensing motion that is caused by the mechanical interference between the driving and the sensing modes. In order to eliminate the coupling effect between driving and sensing modes and achieve a low zero-point output drift, the gyroscope should be designed to have the decoupled structure, which reduces the degree of the coupling, *r*, in Eq. (14).

**Figure 6.**
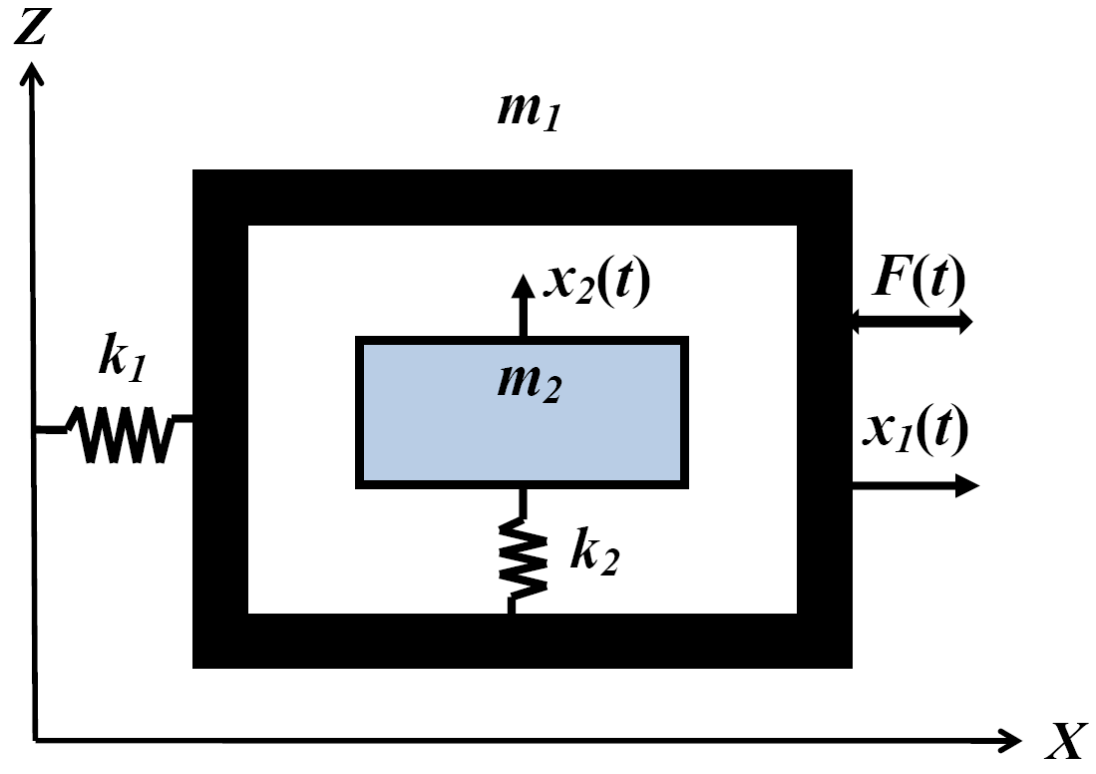
Modeling of the decoupled gyroscope.

To decrease mechanical coupling, various structures have been proposed where the drive and sense motions are independent [5-7]. We propose a new decoupled gyroscope to prevent the mechanical coupling. The mechanical modeling of the decoupled gyroscope is shown in Fig. 6, corresponding to *k*=0 at the coupled modeling in Fig. 2.

**Figure 7.**
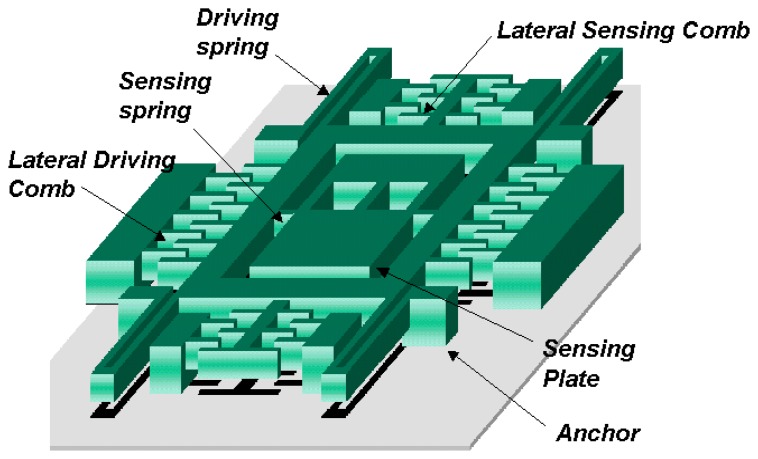
The schematic of a new gyroscope.

The schematic of the new decoupled vertical gyroscope is shown in Fig. 7. Theoretically decoupled design in Fig. 6 has been accomplished in the new gyroscope by dividing the driving and sensing modes in comb structures. The key factor for the decoupled gyroscope is to eliminate the transmission of the driving input into the sensing motion. The proposed comb structures dramatically reduce the structural coupling by locating the rotational suspension of the sensing plate at the nodal point of the driving vibration mode. The stiffness and mass of the driving structure in Fig. 7 correspond to the theoretical models *k*_1_ and *m*_1_ in Fig. 6, respectively. Also the rotational stiffness and mass of the sensing plate are *k*_2_ and *m*_2_ in Fig. 6. The gyroscope has 4-driving springs suspending the whole mass, the driving comb electrodes, and the driving-sensing comb electrodes. Under the inner mass, there are the bottom electrodes that sense the tilting of inner mass. The outer frame is connected to the substrate by 4-driving springs. The mass is divided into two parts, that is to say, the inner mass and the outer frame.

The inner mass and the outer frame that is around the inner mass are connected with two torsional sensing springs. When the driving voltage applies on the driving comb electrode on the side of the outer frame, the mass oscillates along x-axis with the driving frequency. The gyroscope rotates about y-axis, which generates Coriolis force along the z-axis. The generated Coriolis force makes the asymmetry inner mass tilt, which makes the capacitance between the inner mass and the bottom electrode change.

**Figure 8.**
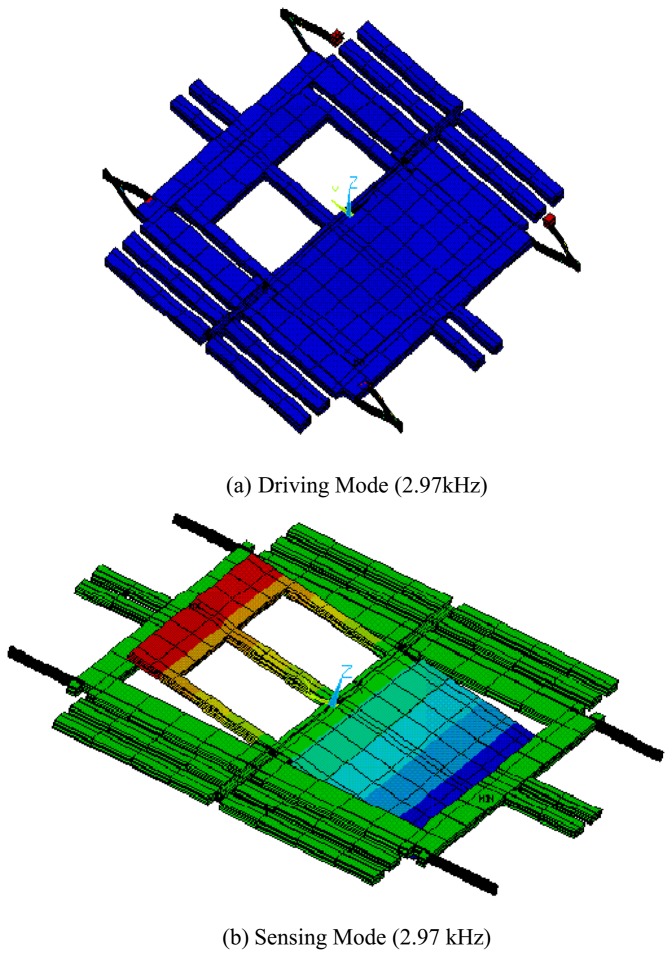
FEM Modal Simulation (a) Driving Mode (2.97kHz) (b) Sensing Mode (2.97 kHz).

**Figure 9.**
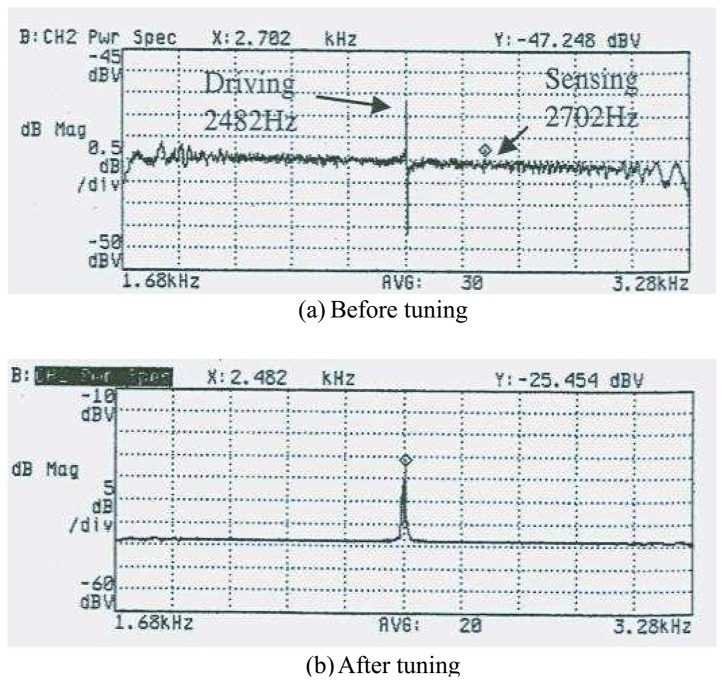
The experimental results for the gyroscope (a) Before tuning (b) After tuning.

FEM modal simulation is used for calculating the frequencies and the deformed shapes of the driving and the sensing modes. Figure 8 shows the result of FEM simulation by commercial code ANSYS. The frequencies of the driving and the sensing mode are same (2.97kHz) as shown in Fig. 8. The result of simulation confirms that there is no interference between the driving and the sensing modes because of the decoupled spring structure. However, it should be noted that the final fabricated structure has the approximate difference of 220 Hz between the driving and the sensing mode frequencies. In addition, they have shifted about 300Hz from the frequencies of the simulation. Figure 9 shows the results from the experiment. The frequencies of the driving and the sensing modes are 2.48kHz and 2.70kHz, respectively.

## 4. Experimental Section

The dynamic characteristics of zero-point output caused by structural coupling have been analyzed. The elliptic trajectory of zero-point output for the coupled gyroscope, which was analytically derived in this paper, has been verified through the results from the experimental measures by Mochida *et al*[5].

The decoupled model suggested theoretically in the previous section should be slightly changed as shown in Fig. 9. The sensing frequency is designed about 220Hz higher than the driving frequency because of the electrostatic tuning and the fabrication tolerance. The mismatching of the driving and the sensing frequencies by fabrication tolerance and/or other reason resulted in low sensitivity. However, the driving and the sensing frequencies could be made to approach each other by changing the effective stiffness of the sensing spring by using a dc-bias voltage on the sensing electrode.

The surface micromachining technology using LPCVD poly silicon has been generally used to fabricate the vertical gyroscope with the bottom electrode. However, the poly crystalline silicon structure would be unstable due to the residual stress and the stress gradient. Besides, it is difficult to deposit the LPCVD poly silicon with over 10 μm-thick repeatedly.

In the Reverse Surface micromachining, the single crystalline silicon structure with high aspect ratio could be fabricated without residual stress or stress gradient. The processing sequences are as follows briefly:

To begin with, a highly doped n-type wafer (0.01∼0.05 Ωcm) is prepared for the structure layer. On the structure wafer, TEOS is deposited for the sacrificial layer, and then the anchor pattern is formed. The LPCVD poly silicon layer is deposited and formed for the bottom electrode and the feed-through for the interconnection. On the LPCVD poly silicon layer, SI_3_N_4_, SiO_2_ and the 10μm-thick epitaxial poly silicon layers are passivated for the insulation and the substrate wafer bonding.

**Figure 10.**
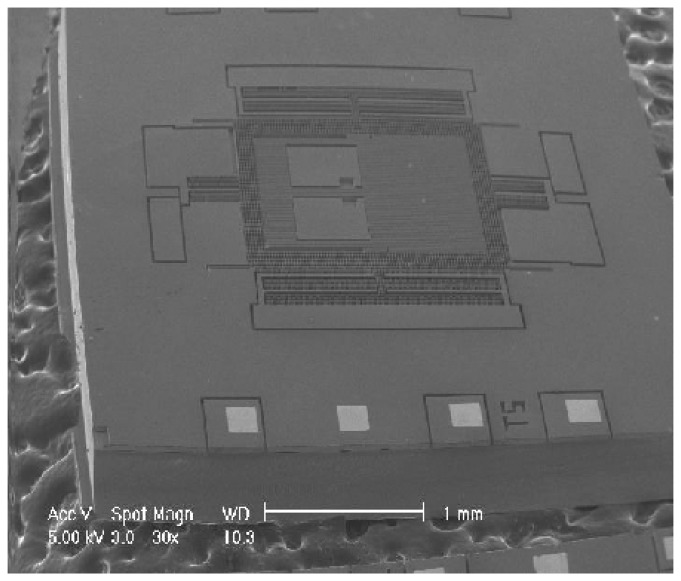
The fabricated decoupled gyroscope.

The epitaxial poly silicon layer is polished for SDB (Silicon Direct Bonding). After the substrate wafer is bonded on the polished epi layer to handle, the structure layer is lapped and polished to 40μm thickness with CMP (Chemical Mechanical Polishing). For the electric pad, Cr/Au is deposited and patterned. The gyroscope structure is formed with deep etcher, ICP RIE. Finally, the sacrificial layer is removed by BOE (Buffered Oxide Etcher) solution to release the gyroscope structure. Fig. 10 shows the fabricated decoupled gyroscope.

## 5. Conclusions

The zero-point output due to the structural coupling between the reference and the sensing vibrations of vertically coupled structures were theoretically studied. A new design of a vertically decoupled gyroscope was proposed based on the dynamic characteristics of the structurally coupled system. To reduce coupling effects, the decoupled gyroscope is designed to have the unconstrained springs for the driving and the sensing modes. And the gyroscope was fabricated with the new mixed micromachining having the 40μm-thick single crystalline silicon structure without residual stress or stress gradient. The micro gyroscope could be used for a hand wobble sensor for camcorders, or a 3-dimansional mouse, and in the future it is expected to be applied to the yaw rate and an acceleration sensor for a vehicle dynamic control or a navigation system.

## Figures and Tables

**Table 1.** Shapes and turning directions of ellipse depending on reference vibration frequency.

**Reference frequencies**	**Shapes**	**directions**
0 < *ω*^2^ < 1	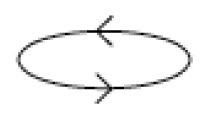	Forward
*ω*^2^ ≈ 1	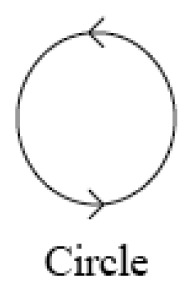	Forward
1 < *ω*^2^ <(1 + *r*^2^)	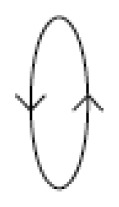	Forward
*ω*^2^ ≈ 1 + *r*^2^	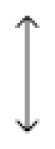	·
(1 + *r*^2^) < *ω*^2^ < (1 + 2*r*^2^)	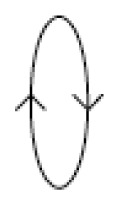	Backward
*ω^2^* ≈ 1 + 2*r*^2^	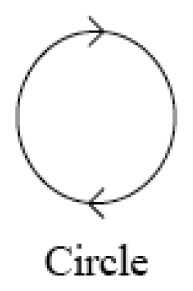	Backward
*ω*^2^ > 1 + 2*r*^2^	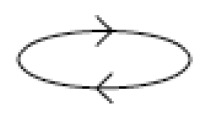	Backward
